# CHD4-induced up-regulation of ERα activity contributes to breast cancer progression

**DOI:** 10.1016/j.gendis.2023.101108

**Published:** 2023-09-15

**Authors:** Aman Sattout, Xiaomin Yu, Zhuo Sun, Yanan Li, Yulin Li, Shujing Li, Wei Huo, Huijian Wu

**Affiliations:** a School of Bioengineering & Key Laboratory of Protein Modification and Disease, Liaoning Province, Dalian University of Technology, Dalian, Liaoning 116024, China; b Department of Oncology, Central Hospital Affiliated with Dalian University of Technology, Dalian, Liaoning 116089, China

The estrogen signaling system is a crucial regulator of metabolic and physiological processes. However, abnormal activation of estrogen signaling may play a role in breast cancer initiation and progression. Crucial to this pathway is the interaction between estrogen receptor alpha (ERα) and various co-transcription activators.^1^ Although numerous studies have investigated ER coregulators, the protein–protein interaction networks of ERα are not fully understood. Recent research has shown that high chromodomain helicase DNA-binding 4 (CHD4) expression is linked to poor prognosis in various cancers.^2^^,^^3^ In this study, we demonstrated that both CHD4 and ERα contribute to breast cancer progression while providing evidence of the regulatory processes and functional interplay between these two proteins.

CHD4 is important to transcriptional regulation and cellular mechanisms.^2^ CHD4 mRNA is up-regulated in all breast cancer subtypes, including ERα-positive breast cancer cells, suggesting that CHD4 expression may be a useful diagnostic tool for such patients.^3^ These prior results are consistent with our analysis of human data from the cBioPortal database. We found that high *CHD4* expression was positively correlated with *ESR1* expression (Fig. 1A) and ERα target *Cyclin D1* (*CCND1*) expression (Fig. 1B). Luciferase assay was used to verify the effect of CHD4 on ERα transcriptional activity. Because ERα regulates estradiol (E2) response, we compared E2-independent and -dependent effects of CHD4 overexpression and knockdown on ERE-luc in MCF-7 cells (Fig. S1A). CHD4 overexpression up-regulated reporter activity for ERE-Luc and *CCND1* promoter Luc in T47D and MCF7 cells, reflecting a positive influence of CHD4 on ERα transcriptional activity (Fig. 1C and D). Silencing CHD4 with specific shRNAs reversed this effect (Fig. 1E, F).

**Figure 1:**
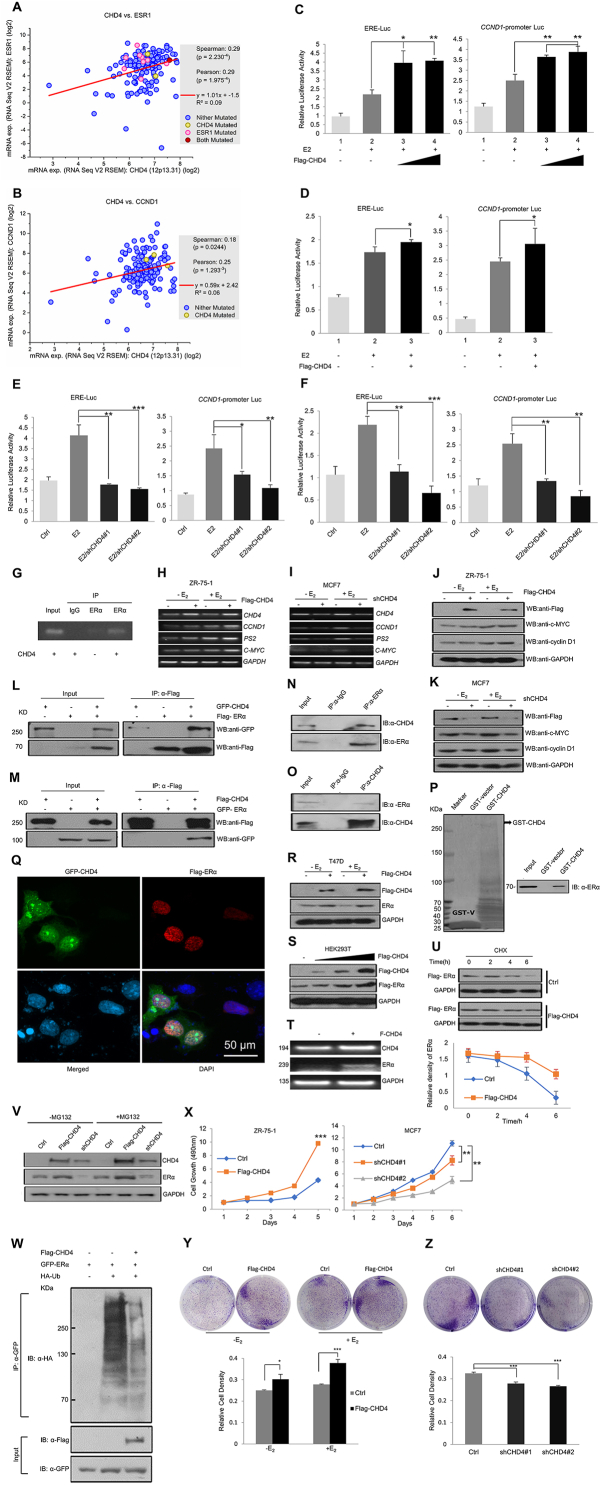
The role of CHD4 in ERα-positive breast cancer cell growth. **(A, B)** Genetic algorithm (GA) analysis of human data from cBioPortal revealed that CHD4 mRNA was positively correlated with *ESR1* (A) and ERα target gene *CCND1* mRNA (B) in patients with ERα-positive breast cancer. **(C, D)** Cells transfected with the indicated plasmids were starved for 12 h in a free phenol-red medium treated with charcoal-stripped serum (CSS). The medium was incubated for 16 h with or without 100 nM estradiol (E2). Luciferase reporter assay was then conducted on the cells, revealing that CHD4 increased ERα transcriptional activity on ERE-luc and *Cyclin D1*-promoter luc in T47D (C) and MCF-7 cells (D). **(E, F)** Knockdown of endogenous CHD4 decreased ERα activation on ERE-luc and *CCND1*-promoter luc in T47D (E) and MCF-7 cells (F). Data are presented as mean ± SD. ^∗^*P* < 0.05, ^∗∗^*P* < 0.01, ^∗∗∗^*P* < 0.001. **(G)** ChIP assays of CHD4's effects on ERα-*CCND1* promoter interaction in MCF7 cells. Cross-linked chromatin was extracted from MCF7 cells and subjected to immunoprecipitation (IP) with anti-IgG or anti-ERα. Purified DNA was then analyzed with RT-PCR. **(H, I)** Cells transfected with the indicated plasmids were treated with or without 100 nM E2 as in C and D. Total RNA was extracted and transcribed into cDNA and then subjected to RT-PCR. ERα-target genes *pS2*, *c-MYC*, and *CCND1* mRNA levels were measured under forced CHD4 expression in ZR-75-1 (H) and under endogenous CHD4 knockdown in MCF7 cells (I). **(J, K)** Cells transfected with the indicated plasmids were treated with or without 100 nM E2 as in C and D. Western blotting (WB) was used to assess CCND1 and c-MYC protein levels under CHD4 overexpression in ZR-75-1 (J) and under shCHD4 (CHD4 silencing) in MCF7 (K). **(L, M)** HEK293T cells were transfected with the indicated plasmids for 36 h and then subjected to co-immunoprecipitation (Co-IP) with Flag antibody, followed by WB with GFP antibody. Flag-ERα specifically precipitated GFP-CHD4 (L); Flag-CHD4 specifically precipitated GFP-ERα (M). **(N, O)** After reaching 90% density, cells were subjected to Co-IP with anti-ERα antibody, followed by WB with anti-CHD4 antibody in MCF7 cells (N), or were subjected to Co-IP with anti-CHD4 antibody, followed by WB with anti-ERα antibody in ZR-75-1 cells (O). The control was Co-IP using an anti-IgG antibody. **(P)** GST alone and GST-CHD4 fusion proteins were expressed in *Escherichia coli* BL21 cells and purified using the Pierce GST Spin Purification Kit. Purified GST-CHD4 fusion protein (BAIT) was immobilized on the Pierce Spin Column and then preyed endogenous ERα from MCF7 cell lysate. WB of GST pull-down assay showed the interaction between CHD4 and ERα. **(Q)** MCF-7 cells were overexpressed with GFP-CHD4 and Flag-ERα for 24 h. ERα was stained with anti-Flag antibody and tetramethylrhodamine isothiocyanate (TRITC)-conjugated anti-rabbit IgG; CHD4 was visualized with green fluorescence. Nuclei were detected with 4,6-diamidino-2-phenylindole (DAPI) staining. Immunofluorescence indicated the colocalization of CHD4 (green) and ERα (red) in the nuclei (blue) of MCF7 cells. **(R, S)** After transfecting cells with CHD4, WB demonstrated that endogenous ERα protein was up-regulated in T47D cells with and without E2 (R). With exogenous ERα in HEK293T cells (S), the effect of CHD4 was dose-dependent. **(T)** ERα mRNA levels with CHD4 overexpression in MCF7 cells shown by RT-PCR. **(U)** HEK293T cells transfected with Flag-ERα only, or with combined Flag-ERα and Flag-CHD4, were administered 10 μg/mL cycloheximide (CHX) for indicated times (in hours) before being subjected to WB. The line graph shows the relative intensity of ERα protein at different times normalized to GAPDH (bottom). **(V)** MCF7 cells transfected with the indicated plasmids for 24 h were treated with or without 10 μM proteasome inhibitor MG132 for 8 h. Samples were subjected to WB with the indicated antibodies. **(W)** HEK293T cells were transfected with the mentioned plasmids for 36 h and then subjected to Co-IP with GFP antibody, followed by WB with HA antibody. Co-IP assay showed the effect of CHD4 on exogenous ERα ubiquitylation in transfected HEK293T cells treated with 10 μM MG132 for 8 h. **(X)** Proliferation curves of CHD4-overexpressing or CHD4-knockdown cells. Cells were seeded in 96-well plates (2500 cells per well) and then visualized using 3-(4,5-dimethylthiazol-2-yl)-2,5-diphenyltetrazolium bromide (MTT) assays to show the effect of CHD4. **(Y, Z)** Cells transfected with the indicated plasmids were seeded in 6-well plates (3000 cells per well) and then treated with and without E2 in ZR-75-1 cells (Y) or without 100 nM estradiol (E2) in MCF7 cells (Z). After 6 days of growth, colony formation was assessed using crystal violet staining. Bar graphs depict relative cell intensity (bottom). Data are presented as mean ± SD. ^∗^*P* < 0.05, ^∗∗^*P* < 0.01, ^∗∗∗^*P* < 0.001.

We further speculated that CHD4 may have a role in ERα binding to a promoter. Chromatin immunoprecipitation (ChIP) assay revealed an ERα increase at the *CCND1* promoter in MCF7 cells following CHD4 overexpression (Fig. 1G), confirming our prediction.

To verify these findings, we analyzed ERα-targeted genes in ZR-75-1 and MCF7 cells, including *pS2*, *CCND1*, and *c-MYC*. CHD4 overexpression increased mRNA and protein levels of ERα-targeted genes (Fig. 1H, J), whereas CHD4 knockdown down-regulated them (Fig. 1I, K). Thus, the evidence suggests that CHD4 enhances ERα transcriptional activity in ERα-positive breast cancer cells.

Before assessing CHD4 and ERα interactions in breast cancer, we conducted Western blot analysis to determine CHD4 and ERα protein levels in various breast cancer cell lines and HEK293T cells. CHD4 and ERα protein concentrations were strongly correlated in ERα-positive breast cancer cells (Fig. S2A). Next, co-immunoprecipitation (Co-IP) was performed using HEK293T (Fig. 1L, M), MCF7, and ZR-75-1 (Fig. 1N, O) cells. We also conducted Co-IP assays in the presence and absence of E2 (Fig. S2B). Both experiments revealed that CHD4 and ERα interact with one another regardless of E2 treatment. The physical interaction was then confirmed with GST pull-down (Fig. 1P) and immunofluorescence (Fig. 1Q). Together, these data showed that CHD4 interacts with ERα in human breast cancer cells and were both located in the nucleus.

We measured the effect of CHD4 on ERα protein levels to understand their relationship. Western blot analysis revealed that CHD4 up-regulation led to a corresponding increase in ERα protein concentrations (Fig. 1R, S). Conversely, shRNA silencing of CHD4 dramatically decreased ERα (Fig. S3A, B). To determine whether CHD4 regulation involved altering ERα mRNA expression or protein degradation, we first measured ERα mRNA with RT-PCR; CHD4 overexpression increased ERα mRNA levels (Fig. 1T). Second, we analyzed the half-life of exogenous ERα protein with and without CHD4 after treating cells with cycloheximide, a protein biosynthesis inhibitor. The results indicated that CHD4 overexpression changed ERα protein level and increased its half-life (Fig. 1U).

In the presence of proteasome inhibitor MG132, CHD4 elevated endogenous ERα protein levels above the levels without MG132 (Fig. 1V). Moreover, CHD4 decreased ERα ubiquitination (Fig. 1W). Taken together, these results suggested that CHD4 inhibits ERα ubiquitin-dependent degradation to stabilize ERα protein synthesis. Thereby, CHD4 increases ERα protein levels through two pathways' gene expression and ERα protein stability.

The ERα-CHD4 relationship and the pivotal role of ERα in promoting the growth of hormone-responsive breast tumor cells prompted us to examine the biological function of CHD4 in the context of ERα. Our proliferation assay revealed that forced CHD4 expression significantly increased both E2-dependent and -independent proliferation of ZR-75-1 cells from control levels. Furthermore, CHD4 knockdown in MCF7 cells lowered cell viability (Fig. 1X; Fig. S4A). To validate these data, we performed a colony formation experiment. CHD4 overexpression increased colony count from control numbers. Conversely, CHD4 knockdown reduced colony formation (Fig. 1Y, Z).

ERα interactions with coregulator proteins are responsible for ligand-independent and ligand-dependent signaling that cause breast cancer formation and progression. Our study discovered that CHD4 promoted ERα-positive breast cancer cell proliferation and up-regulated ERα protein in the absence of E2. Moreover, we demonstrated that CHD4 induces ligand-independent activation of ERα. Furthermore, we showed that the presence of E2 enhanced CHD4 effects on ERα activation. This finding corroborates previous research in ovine endothelial cells: ERα is active in the absence of E2, but E2 exposure gradually increases ERα transcriptional activity, with long-term E2 treatment (6 h or 12 h) up-regulating ERα protein.^4^^,^^5^

In summary, we investigated the ERα-CHD4 interaction and its association with breast cancer progression. Our findings demonstrated that CHD4 exhibits oncogenic properties, as evidenced by its overexpression up-regulating ERα through two pathways, increasing the mRNA of ERα and decreasing its protein ubiquitin-dependent degradation in ERα-positive breast cancer. Conversely, CHD4 knockdown resulted in the opposite effect. Moreover, CHD4 increased ERα-mediated growth of ER-positive breast tumor cells *in vitro*. Besides, we found that *ESR1* promoter has CHD4 binding site between −499 bp and −494 bp; therefore, we speculated that CHD4 may up-regulate the mRNA level of ERα via binding to the *ESR1* promoter. This area needs further investigation.

In conclusion, these findings provide novel insight into the molecular mechanism underlying ERα regulation and highlight a possible role for CHD4 as a biomarker that can benefit the development of hormone therapy for ERα-positive breast cancer. This study highlights the importance of continued investigation into the complex biological processes underlying ERα-positive breast cancer.

## Author contributions

HW and WH conceived and designed the experiments. AS, XY, and ZS carried out the experiments. AS, XY, Yanan L, and SL analyzed the data. AS, XY, Yulin L, and SL contributed reagents/materials/analysis tools. HW and AS wrote the manuscript. All authors read and approved the final manuscript.

## Conflict of interests

The authors declare that they have no conflict of interests.

## Funding

This work was supported by a grant from the 10.13039/501100001809National Natural Science Foundation of China (No. 81872263 to HW).

## Data availability

Because no datasets were collected or analyzed during the current study, data sharing is not applicable to this publication.
