# Supplementary material for: CHD4-induced up-regulation of ERα activity contributes to breast cancer progression
Source: Genes Dis. 2023 Sep 15;11(3):101108. doi: 10.1016/j.gendis.2023.101108 (PMC10825269; doi:10.1016/j.gendis.2023.101108)
Supplement: Multimedia component 1 [file mmc1.docx]

Supplementary Figures:

Supplementary Figure 1:

**A**


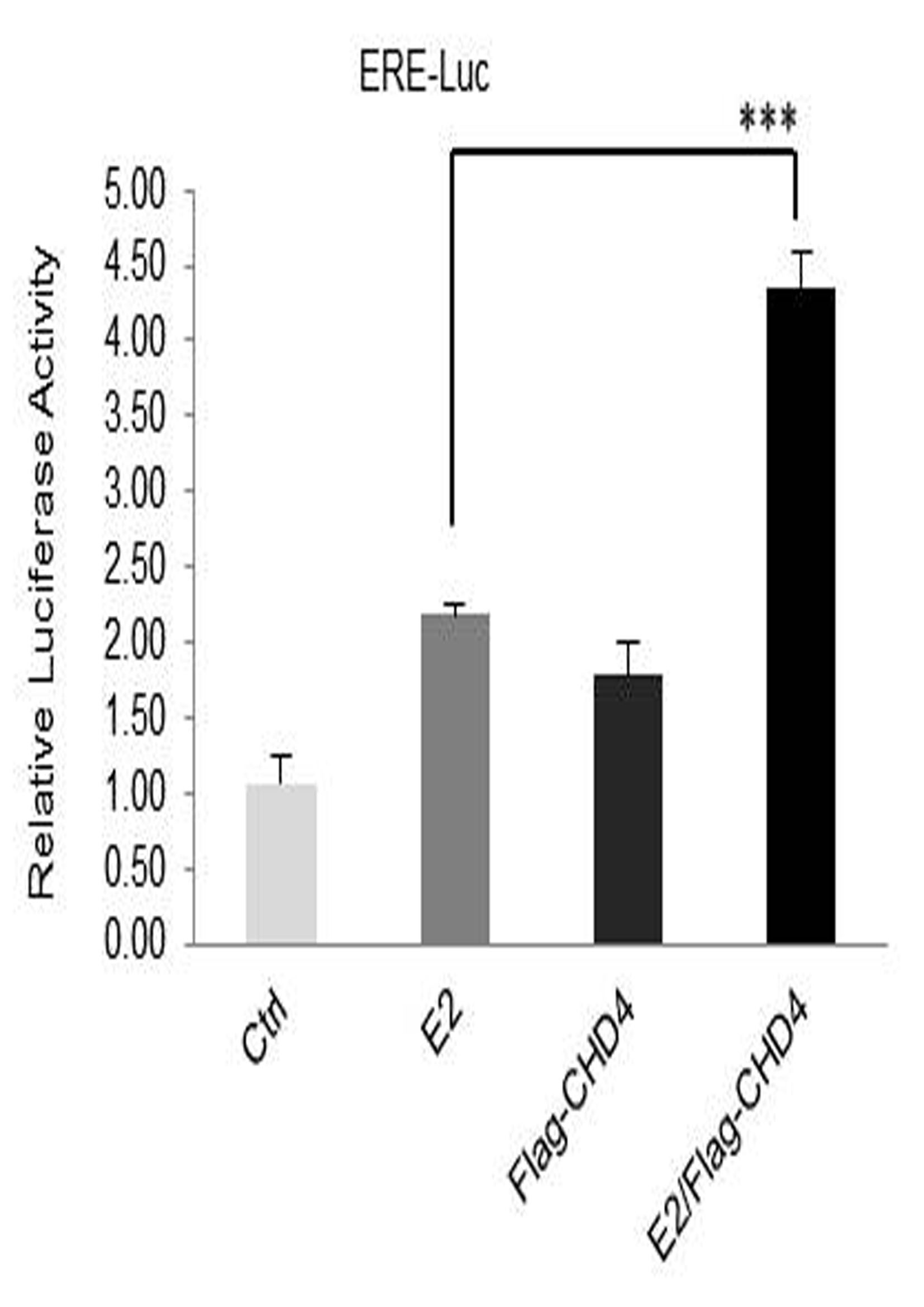

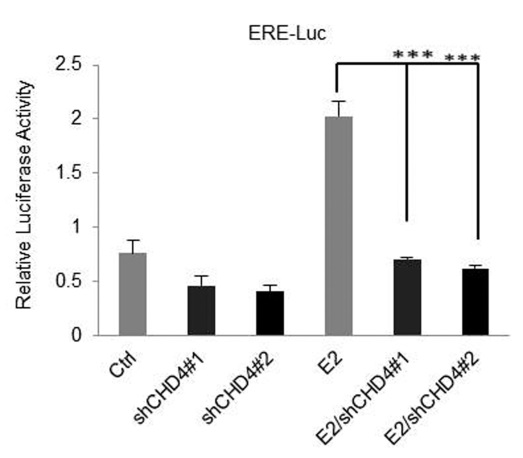


**A** MCF7 Cells transfected with the indicated plasmids were followed either with or without 100 nM estradiol (E2) for 16 h. CHD4 expression was knocked down with two different short hairpin RNA (shRNA), shCHD4#1 and shCHD4#2. Luciferase reporter assay shows E2-independent compared to E2-dependent effects of CHD4 overexpression and knockdown on ERE-luc.

Supplementary Figure 2:


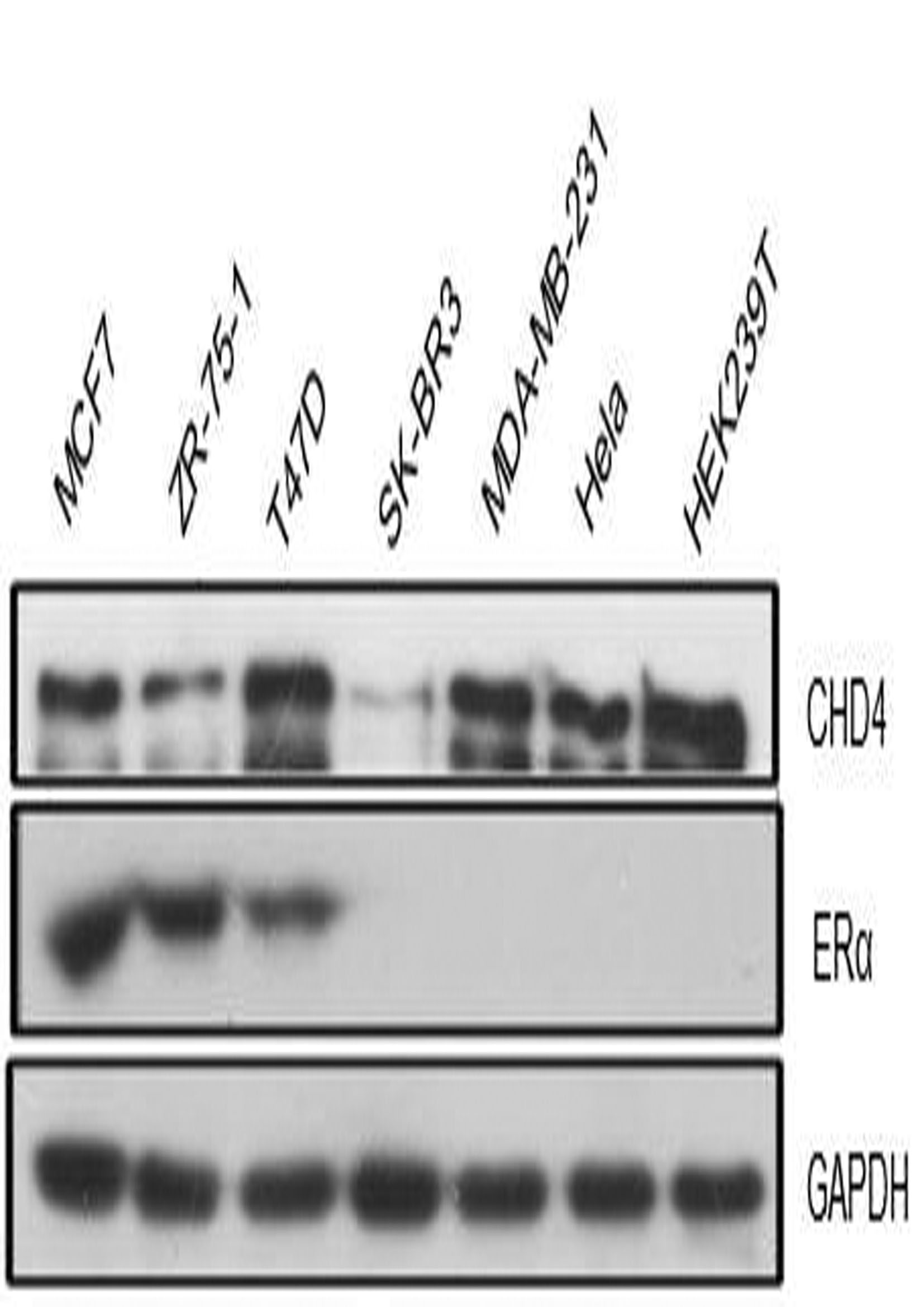


**A**

**A** After reaching 90% density, cells were subjected to western blotting and detected CHD4 and ERα protein levels in the mentioned cell lines.


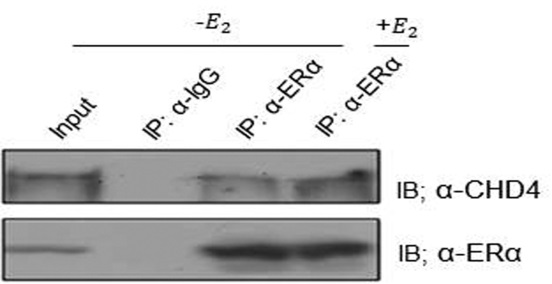


**B**

**B** MCF7 cells were treated with or without 100 nM estradiol (E2) for 16 h and then subjected to Co-IP with anti-ERα antibody, followed by western blotting with anti-CHD4 antibody. The control was Co-IP using anti-IgG antibody.

Supplementary Figure 3:

**A**


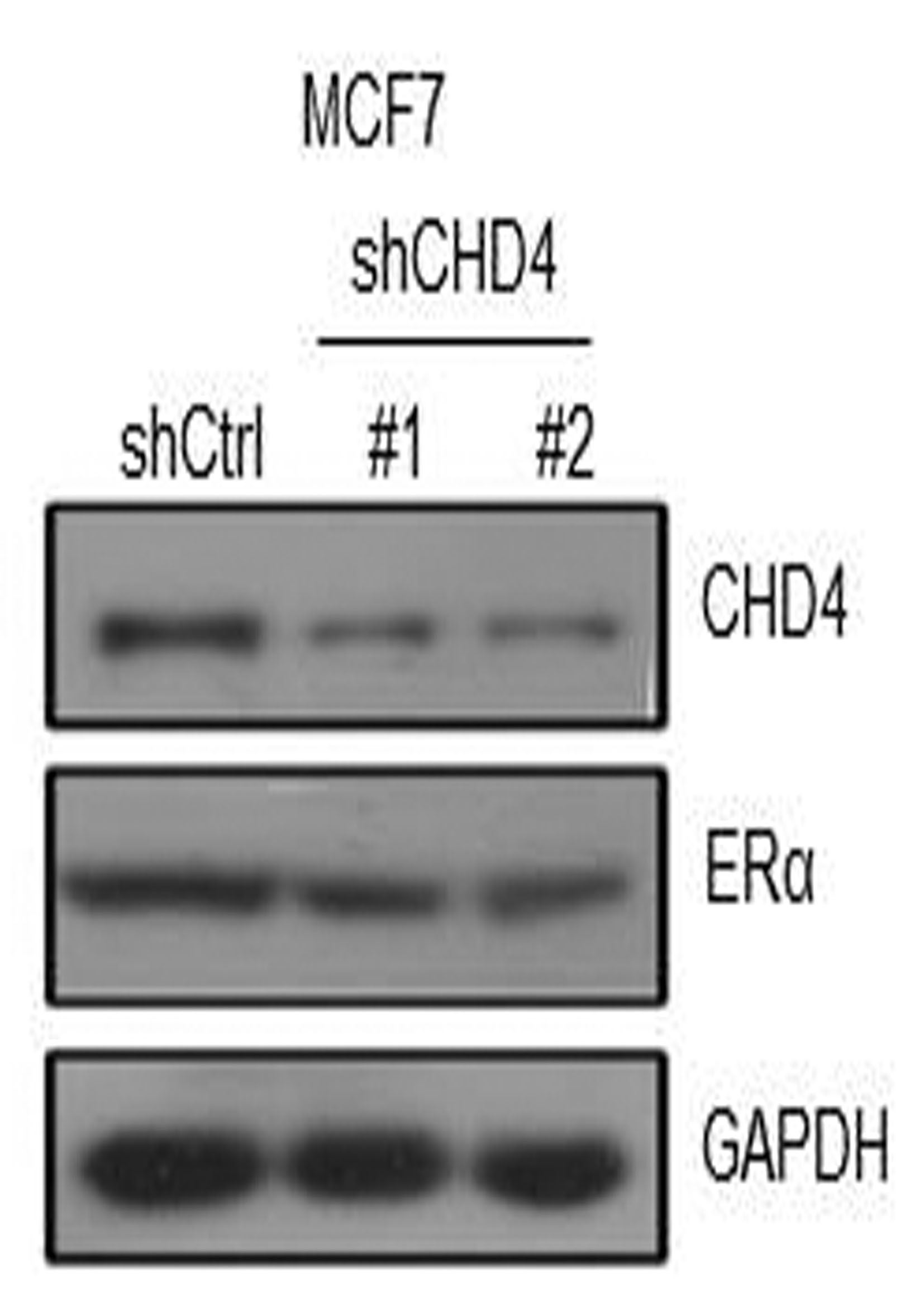

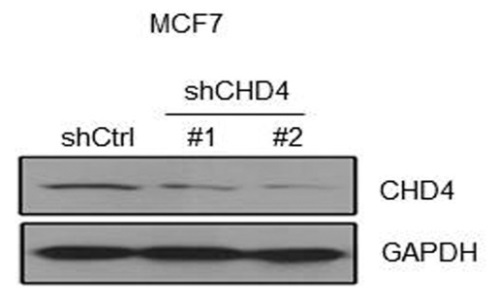


**
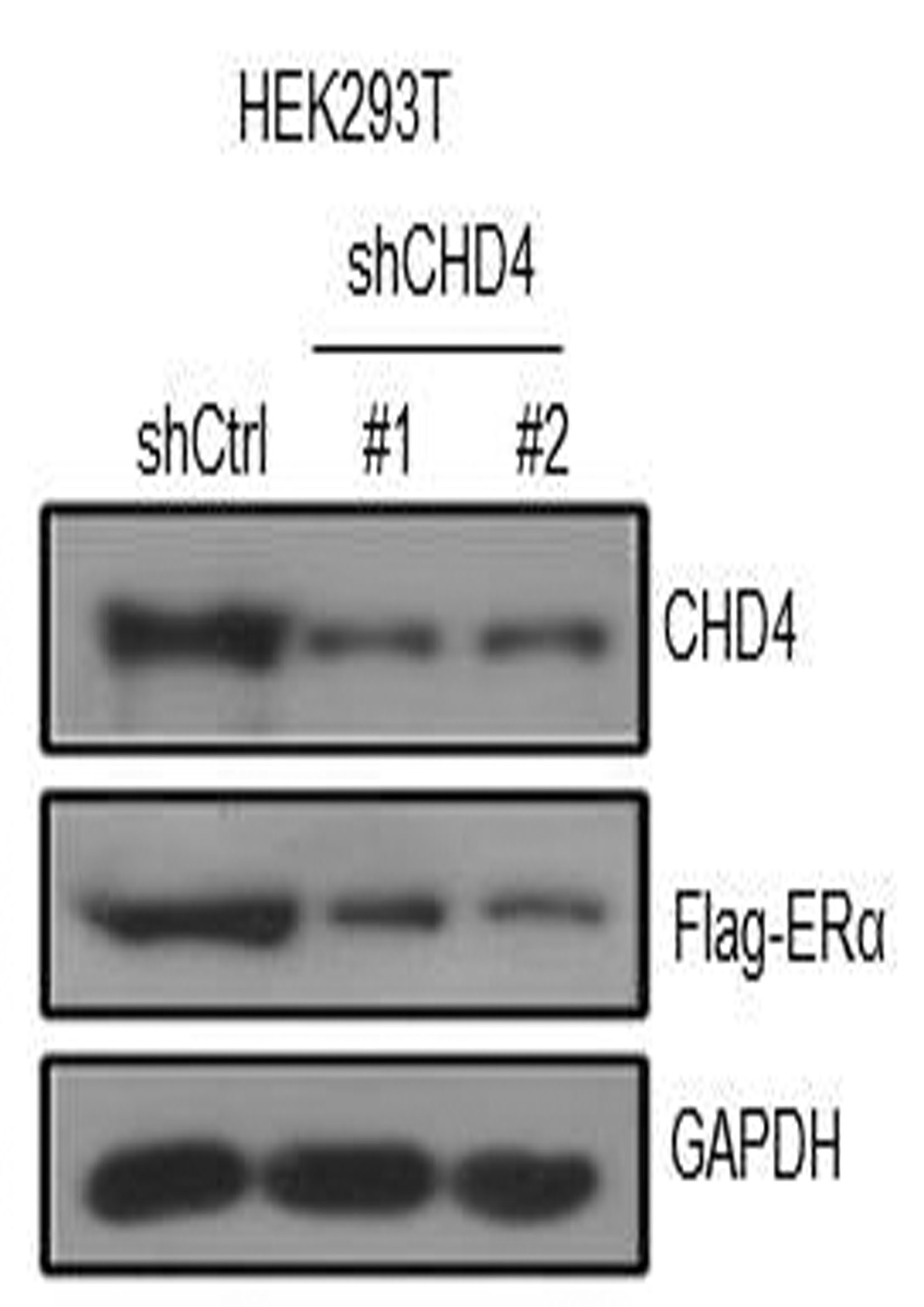

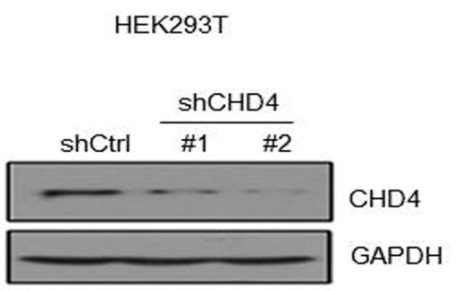
**

**B**

**A&B** After transfecting cells with the indicated plasmids for 36 h, western blotting demonstrated that shCHD4 may reduce endogenous ERα protein levels in MCF7 cells **(A)** and exogenous ERα in HEK293T cells **(B)**.

Supplementary Figure 4:

**A**


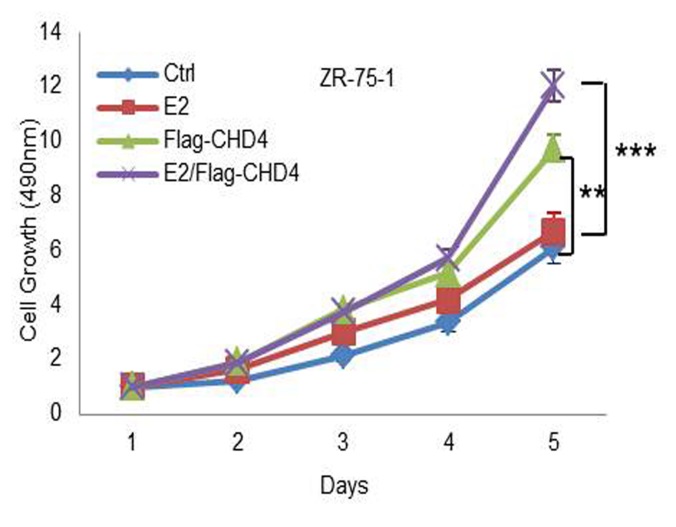


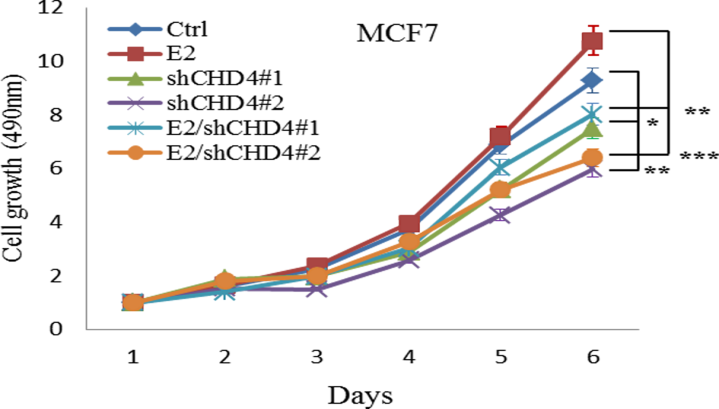


**A** Proliferation curves of CHD4-overexpressing or CHD4-knockdown cells using two different short hairpin RNA, shCHD4#1 and shCHD4#2 were visualized using the MTT assay and showed the effect of CHD4 on E2-dependent compared to E2-independent cell proliferation.

**Materials and methods**

**Cell culture and cell lines**

We used HEK293T, MDA-MB-231 and HeLa cells in our research and our earlier studies.^1,2^ The T47D, MCF7, ZR-75-1, and SK-BR-3 breast cancer cells were generously offered by Professor Wei Cheng (Dalian Medical University).

The cell lines were cultured in a humidified environment set to 5% CO2, 37 °C.

**Plasmids and transfection**

The pGL3-Basic vector was used to subclone the CCND1-Luc sequence, which contained the promoter region from 960 to 136. (Promega, Madison, WI, USA). Dr. WeiGuo Zhu provided full-length human Flag-CHD4, GFP-CHD4, and GST-CHD4 plasmids at the Peking University Health Science Center, China). The gene expression vectors of CHD4 small hairpin RNA (shRNA) were created using the vector pRNATU6.1 and the sequence as follows:5′ - CCTTACTAGAATTGGTGTTAT -3’ (#1), 5′- GCTGCTGACATC CTATGAATT -3’ (#2). The results of western blotting verified the effects of shRNA. Full-length ERα has been previously described.^3^

When the cells reached approximately 70% confluence, Lipofectamine 2000 (Invitrogen, Auckland, New Zealand) was used to transfect them with the appropriate plasmids.

**Antibodies and reagents**

Mouse anti-Flag M2 (1:3000, F1804) and anti-Flag rabbit (1:500, F7425) were purchased from Santa Cruz Biotechnology. Anti-GFP (1:3000, GTX113617), anti-GAPDH (1:6000, TA802519), and anti-GST (1:3000, ABN116) were purchased from GeneTex, ORIGENE, and Millipore, respectively. Santa Cruz Biotechnology provided a mouse monoclonal antibody against ERα (F-10) for this study (Santa Cruz), and Abcam (ab240640) provided a rabbit monoclonal antibody against CHD4. anti-c-MYC (1:500, WL01781) and anti-CCND1 (1:500, WL01435a) were purchased from Wanlei.

Bimake (Houston, USA) provided protein A/G mix magnetic beads (B23202) and an anti-FLAG affinity gel (B23102).

MG132 was obtained from Selleck and Cycloheximide was obtained from Sigma.

**Luciferase reporter assay**

A 24-well plate was filled with cultured cells a 1 × 105/well. The cells were transfected with the relevant plasmids using Lipofectamine 2000 after 24 h. After 24 h of transfection, a luciferase assay was conducted on the cells according to the manufacturer's specifications.

**RNA extraction and real-time PCR**

RNAiso reagent (Takara) was used to extract total RNA after 48 h from the appropriate plasmid-transfected MCF7 and ZR-75-1 cells. The RNA was transcribed into cDNA, and the results were detected by RT-PCR.

Table 1. Primers for RT-PCR testing

| Primer name | Primer sequence |
| --- | --- |
| ESR1 (forward) | 5’-actcgctactgtgcagtgtgcaat-3’ |
| ESR1 (reverse) | 5’-cctcttcggtcttttcgtatccca-3’ |
| CHD4 (forward) | 5’-caaagtggcccagtatgtgg-3’ |
| CHD4 (reverse) | 5’-agttgacctgtttacggatt-3’ |
| c-MYC (forward) | 5’-agggatcgcgctgagtataa-3’ |
| c-MYC  (reverse) | 5’-tgcctctcgctggaattact-3’ |
| CCND1 (forward) | 5’-gctgctcctggtgaacaagc-3’ |
| CCND1 (reverse) | 5’-aagtgttcaatgaaatcgtgcg-3’ |
| pS2 (forward) | 5’- atggagaacaaggtgatctg-3’ |
| pS2 (reverse) | 5’- ccacaattctgtctttcacg-3’ |
| GAPDH (forward) | 5′-gggttgaaccatgagaagt-3′ |
| GAPDH (reverse) | 5′-gactgtggtcatgagtcct-3′ |

**Western blot, Co-IP and ChIP assays**

Co-immunoprecipitation (Co-IP), western blot and ChIP experiments were carried out as explained previously.^4-5^ The ChIP assay was conducted using MCF7 cells, and the CHD4 antibody was used for cell lysate immunoprecipitation. The primers used were 5’-ACTGAGGTCCTGGCAGGTTGCATTC -3’ (sense) and 5’- ACGTAGTGTGTACATAGGATGACCC -3’ (antisense) for *CCND1* promoter.

**Immunofluorescence assay**

An immunofluorescence assay was carried out according to earlier descriptions.^6^

**Cell proliferation assay**

The relevant plasmids were transfected into MCF-7 and ZR-75-1 cells and cultured in 96-well plates (2000 cells per well) for 48 h. MTT was then added to the cells for 4 hours, and this method was repeated for another five days. The growth medium was changed to a new one every two days. We then applied DMSO (dimethyl sulfoxide) and measured the absorbance at 490 nm.

**Colony formation assay**

A colony formation was conducted using MCF-7 and T47D cells (3000 cells per well). After 48 h of transfection, cells were cultured in 6-well plates. Cells were starved for 12 h in free phenol-red medium treated with charcoal-stripped serum (CSS). We altered the medium for 16 h with or without 100 nM estradiol (E2). The cells were then washed twice with ice-cold PBS. Subsequently, the solution of crystal violet was utilized to highlight the cell colonies. Following the cell colonies plates being photographed, DMSO was added to determine the absorption at 450 nm.

**GST pull-down assay**

The GST pull-down test was carried out as stated in our earlier study.^7^ Escherichia coli BL21 (Takara, Dalian, China) was used to produce both GST and GST-CHD4 fusion proteins, which were purified using the Pierce GST Spin Purification Kit (Thermo Scientific). The ERα of MCF-7 lysate cells was preyed upon after the bait (GST-CHD4 protein) was precipitated. on the Pierce Spin Column.

**Statistical analysis**

The statistical analyses were carried out using a two-tailed Student's *t*-test to assess the significance between two groups' results. Every experiment has repeated a minimum of 3 times and the data reported were examined as mean± *s.d.* (standard deviations). The *P*-value (probability value) at either 0.05 or less was considered statistically significant.

**References**

1. Li X, Li S, Li B, et al. Acetylation of ELF5 suppresses breast cancer progression by promoting its degradation and targeting CCND1. *npj Precis Oncol*. 2021:1-15. doi:10.1038/s41698-021-00158-3

2. Aman S, Li Y, Cheng Y, et al. DACH1 inhibits breast cancer cell invasion and metastasis by down-regulating the transcription of matrix. 2021;(September):1-9. doi:10.1038/s41420-021-00733-4

3. Xu Z, Yang Y, Li B, et al. Checkpoint suppressor 1 suppresses transcriptional activity of ER α and breast cancer cell proliferation via deacetylase. *Cell Death Dis*. 2018. doi:10.1038/s41419-018-0629-3

4. Bi H, Li S, Wang M, et al. SUMOylation of GPS2 protein regulates its transcription-suppressing function. 2014;25. doi:10.1091/mbc.E13-12-0733

5. Ao X, Li S, Xu Z, et al. Sumoylation of TCF21 downregulates the transcriptional activity of estrogen receptor-alpha. 2016;7(18).

6. Bi H, Li S, Qu X, et al. DEC1 regulates breast cancer cell proliferation by stabilizing cyclin E protein and delays the progression of cell cycle S phase. 2015:1-13. doi:10.1038/cddis.2015.247

7. Zhao F, Wang M, Li S, et al. DACH1 inhibits SNAI1-mediated epithelial – mesenchymal transition and represses breast carcinoma metastasis. 2015;(October 2014):1-14. doi:10.1038/oncsis.2015.3
